# Experimental *Listeria*–*Tetrahymena*–*Amoeba* food chain functioning depends on bacterial virulence traits

**DOI:** 10.1186/s12898-019-0265-5

**Published:** 2019-11-22

**Authors:** Valentina I. Pushkareva, Julia I. Podlipaeva, Andrew V. Goodkov, Svetlana A. Ermolaeva

**Affiliations:** 1 Gamaleya Research Centre of Epidemiology and Microbiology, Gamaleya st. 18, Moscow, 123098 Russia; 2 Institute of Cytology, Russian Academy of Sciences, Saint Petersburg, Russia; 3 National Research Centre on Virology and Microbiology, Pokrov, Russia

**Keywords:** Pathogenic bacteria, Free-living protozoa, Host–parasite interactions, Food web, *Listeria*, *Amoeba*

## Abstract

**Background:**

Some pathogenic bacteria have been developing as a part of terrestrial and aquatic microbial ecosystems. Bacteria are consumed by bacteriovorous protists which are readily consumed by larger organisms. Being natural predators, protozoa are also an instrument for selection of virulence traits in bacteria. Moreover, protozoa serve as a “Trojan horse” that deliver pathogens to the human body. Here, we suggested that carnivorous amoebas feeding on smaller bacteriovorous protists might serve as “Troy” themselves when pathogens are delivered to them with their preys. A dual role might be suggested for protozoa in the development of traits required for bacterial passage along the food chain.

**Results:**

A model food chain was developed. Pathogenic bacteria *L. monocytogenes* or related saprophytic bacteria *L. innocua* constituted the base of the food chain, bacteriovorous ciliate *Tetrahymena pyriformis* was an intermediate consumer, and carnivorous amoeba *Amoeba proteus* was a consumer of the highest order. The population of *A. proteus* demonstrated variations in behaviour depending on whether saprophytic or virulent *Listeria* was used to feed the intermediate consumer, *T. pyriformis*. Feeding of *A. proteus* with *T. pyriformis* that grazed on saprophytic bacteria caused prevalence of pseudopodia-possessing hungry amoebas. Statistically significant prevalence of amoebas with spherical morphology typical for fed amoebas was observed when pathogenic *L. monocytogenes* were included in the food chain. Moreover, consumption of tetrahymenas fed with saprophytic *L. innocua* improved growth of *A. proteus* population while *L. monocytogenes*-filled tetrahymenas provided negative effect. Both pathogenic and saprophytic bacteria were delivered to *A. proteus* alive but only *L. monocytogenes* multiplied within amoebas. Observed differences in *A. proteus* population behaviour suggested that virulent *L. monocytogenes* might slow down restoration of *A. proteus* ability to hunt again and thus restrict the size of *A. proteus* population. Comparison of isogenic bacterial pairs that did or did not produce the haemolysin listeriolysin O (LLO) suggested a role for LLO in passing *L. monocytogenes* along the food chain.

**Conclusions:**

Our results support the idea of protozoa as a means of pathogen delivery to consumers of a higher order and demonstrated a dual role of protozoa as both a “Trojan horse” and “Troy.”

## Background

Terrestrial and aquatic ecosystems include multiple populations of prokaryotic and eukaryotic organisms that function as a whole, united by the complicated food web. Zooplankton including bacterivorous ciliates, flagellates, and amoebas control the bacterial populations in an ecosystem [1, 2]. By consuming bacteria, and then being consumed by predators of higher orders such as metazooplankton, nematodes etc., protozoa represent an important link between aquatic and terrestrial microbial food chains [3]. Unicellular predators consume bacteria both directly and indirectly, supporting bacterial migration from lower to higher orders along the food chains [2, 4, 5].

In recent years, it has become clear that a number of bacteria have developed specific mechanisms of defence against unicellular predators [6–8]. These might be surface modifications or the production of toxic products [8, 9]. Pathogenic bacteria, which are members of terrestrial and aquatic ecosystems, often use virulence factors as a mechanism of defence against microbial predators [10–13]. Incomplete digestion in protozoan phagosomes is also particularly typical for pathogenic bacteria [13–19].

The Gram-positive pathogenic bacterium *Listeria monocytogenes* is widely spread in nature. It has been isolated from multiple natural habitats including soil, sewage, plant debris, plants, and animals such as sheep and cattle, wild deer, boars, small rodents, birds, and fish [20–27]. Soil seems to be an initial point from where *L. monocytogenes* contaminates plants to spread further to herbivorous animals, and soil is a final stop where bacteria come to with animal faeces [21]. Multiple cases of *L. monocytogenes* isolation from fish and molluscs suggested that it is common in aquatic habitats. Interaction of *L. monocytogenes* with other members of terrestrial and aquatic ecosystems should be essential for its prolonged survival in the environment [27–29].

*Listeria monocytogenes* is a facultative intracellular pathogen. The thiol dependent haemolysin listeriolysin O (LLO) is a key virulence factor required for its intracellular survival [30]. Bacteria lacking the LLO-encoding gene *hly* are avirulent in animal models [31]. LLO is a multifunctional protein [32]. Its pore-forming activity facilitates phagosomal membrane disruption and entrance of the pathogen into the cytosol, as well as active invasion of mammalian cells [33, 34]. When *L. monocytogenes* was co-cultivated with the common ciliate *Tetrahymena pyriformis*, LLO-producing bacteria were shown to be toxic for protozoa, caused protozoan encystment, and were more successful than isogenic LLO-lacking bacteria suggesting a leading role of LLO in bacterial survival in the presence of *T. pyriformis* [13]. *L. innocua* is the closest saprophytic species to *L. monocytogenes* that lacks major virulence determinants including LLO [35].

*Tetrahymena pyriformis* and *Amoeba proteus* are two widely spread protozoa that live in fresh water over a wide range of conditions. In the wild, *Tetrahymena* feed on bacteria. In laboratory conditions, axenic *T. piriformis* culture is used as a standard model for studies of interactions between bacteria and unicellular predators [36]. *A. proteus* is among the largest free-living phagotrophic protists with a cell size of up to 800 μm [37]. This is a carnivorous amoeba that preferably grazes on smaller protists such as some ciliates, flagellates, unicellular algae, yeasts, etc. [37].

It was hypothesized that some small soil and freshwater amoebas may act as “Trojan horses” and deliver amoeba-resistant bacteria into the human body [15, 38–40]. However, large free-living amoebas, like representatives of the genus *Amoeba*, might serve as “Troy” themselves when they consume smaller species of protists filled with resistant bacteria. To get evidences on this dual role of protozoa we developed an experimental food chain that included either the pathogenic *L. monocytogenes* or the saprophytic *L. innocua*, the bacteriovorous ciliate *T. pyriformis* and the carnivorous amoeba *A. proteus*. We were interested in a role of pathogenic bacteria in microbial food chain, and particularly, in a difference in microbial food chain dynamics in dependence on whether saprophytic or virulent bacteria comprise the base of the food web.

## Results

### Characterization of *A. proteus* grazing on virulent and saprophytic *Listeria*

**Fig. 1:**
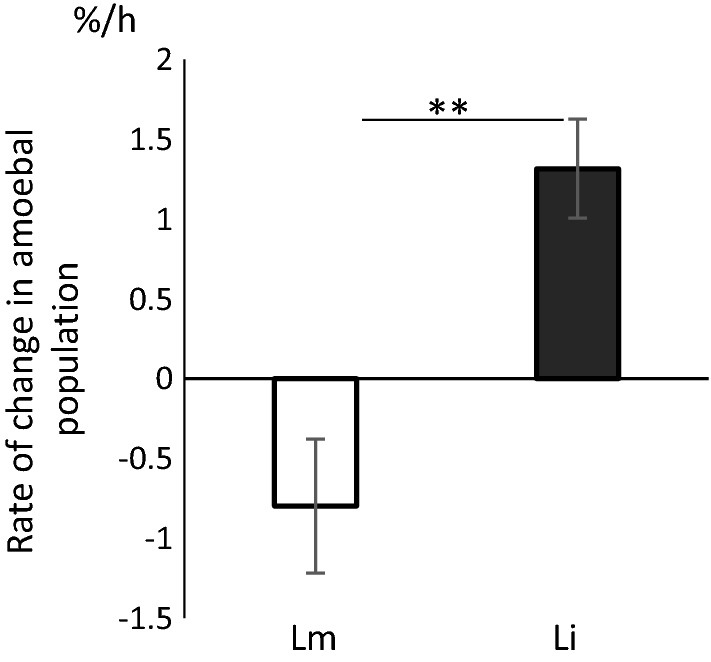
Rates of change in amoebal population stimulated by introduction of bacteria. Lm: virulent *L. monocytogenes*; Li: saprophytic *L. innocua*. Bacteria were added to a starved *A. proteus* culture with multiplicity 1000:1 (bacteria:amoeba) and incubated for 24 h. Amoebas were counted with light microscope at different times points. Rates expressed percentage change per hour were calculated as described in “Methods” section. Positive rates mean an increase in the amoebal population while negative rates mean a decrease. The data represent mean values ± SD from six independent experiments. **p < 0.005

*Listeria monocytogenes* and the saprophytic species *L. innocua* were used to feed *A. proteus*. The starving *A. proteus* population was stable under experimental conditions used and maintained its size constant in the absence of addition of bacteria. There was a gradual increase in the *A. proteus* population grazing on *L. innocua* within 24 h. In contrast, the population of *A. proteus* grazing on *L. monocytogenes* decreased. Rates of changes in *A. proteus* population measured as percentage change per hour were 1.316 ± 0.31% and − 0.798 ± 0.42% in the presence of *L. innocua* and *L. monocytogenes*, respectively (Fig. 1, p < 0.005).

**Fig. 2:**
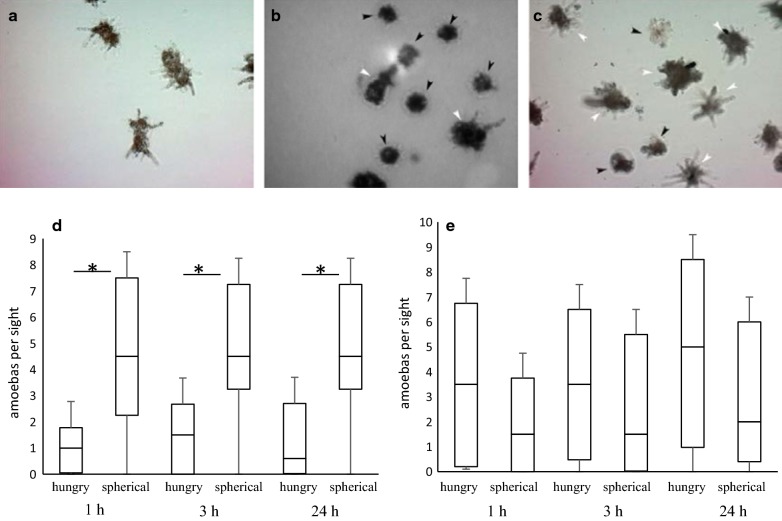
Changes in amoebal morphology caused by grazing on pathogenic and saprophytic *Listeria*. **a** Control (starved) *A. proteus*. **b**, **d**—*A. proteus* fed with *L. monocytogenes*. **c**, **e**—*A. proteus* fed with *L. innocua*. **b**, **c**—amoebas with morphology typical for starved (pseudopodia-possessed) animals are shown with white arrows. Fed amoebas with spherical morphology are shown by black arrowheads. **d**, **e**—counts of amoebas with hungry and spherical morphology per sight at different times points (1, 3 or 24 h) post introduction of the bacterial culture The data from three independent experiments *p < 0.05

**Fig. 3:**
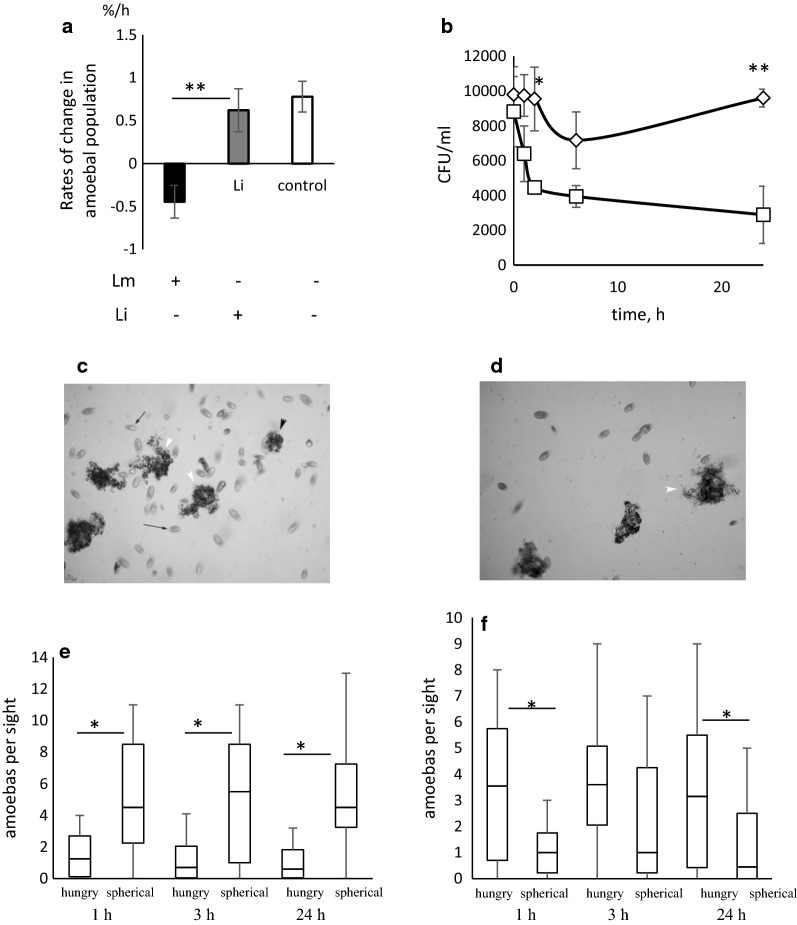
Characteristics of *A. proteus* population changes in the food chain *Listeria*–*Tetrahymena pyriformis*–*A. proteus* dependent of whether saprophytic or virulent *Listeria* was used to feed the intermediate consumer, *T. pyriformis*. **a** Rates of changes in amoebal population stimulated by introduction of *T. pyriformis* which were fed with bacteria. Lm: *T. pyriformis* were fed with virulent *L. monocytogenes*; Li.: *T. pyriformis* were fed with saprophytic *L. innocua*. **b** Intracellular bacteria within of *A. proteus* that grazed *on T. pyriformis* which were fed with the wild type *L. monocytogenes* EGDe strain (diamonds) or with saprophytic *L. innocua* NCTC10288 strain (squares). **c**, **e**—The intermediate consumer *T. pyriformis* fed with *L. monocytogenes*; **d**, **f**—The intermediate consumer *T. pyriformis* fed with L. *innocua*. **c**, **d**—Morphology of *A. proteus* that grazed on *T. pyriformis* which were fed with *L. monocytogenes* EGDe or with *L. innocua* NCTC10288 strain. **e**, **f**—Counts of *A. proteus* with hungry and spherical morphology per viewing field at 1, 3, and 24 h post introduction of *T. pyriformis*. The data from three independent experiment were used. *p < 0.05; **p < 0.01

Addition of both pathogenic and non-pathogenic bacteria caused changes in amoebal morphology (Fig. 2a–c). These changes, including dragging of pseudopodia and the assumption of a spherical, round-shaped form typical for fed amoebas, were correlated with the appearance of digestive phagosomes within amoebas and appeared to be associated with grazing. Amoebas with altered morphology (spherical) were observed as early as 1 h and up to 24 h (the end of the experiment) post bacterial addition. Another portion of the *A. proteus* population retained the morphology typical for hungry cells with characteristic pseudopodia. Enumeration of amoebas with spherical and pseudopodia-possessing (hungry) morphology revealed a statistically significant difference between populations that grazed on virulent and saprophytic bacteria (Fig. 2d, e). In the presence of *L. monocytogenes*, the mean value of morphologically spherical amoebas was 4 times higher than that of hungry amoebas (Fig. 3d, 50th percentile 4.5 vs 1.5 of spherical and hungry cells per viewing field, respectively, after 1 h of co-incubation with bacteria p < 0.05). The ratio of spherical to hungry amoebas changed slowly with time (4.50 vs 1.5 and 4.5 vs 0.6, at 3 and 24 h, respectively; p < 0.05). In the presence of *L. innocua*, the mean number of spherical amoebas was less (1.6–2.5 times) than that of amoebas with hungry morphology since the 1st up to 24 h of co-incubation (50th percentile 1.5 vs 3.5 and 2.0 vs 5.0 for amoebas with spherical and hungry morphology in 1 h and 24 h, respectively, p = 0.09; 0.29).

### Establishing a *Listeria*–*Tetrahymena*–*Amoeba* food chain model

We compared pathogenic and saprophytic bacteria in the model food chain *Listeria*–*T. pyriformis*–*A. proteus*. *T. pyriformis* grazed on pathogenic *L. monocytogenes* or saprophytic *L. innocua* were used to feed starved *A. proteus*, and uninfected tetrahymenas were used as a control.

The total number of amoebas slowly changed over the course of the 24 h experiment. Dynamics of amoebas population fed with *L. monocytogenes*-filled tetrahymenas was negative. In contrast, consumption of tetrahymenas fed with saprophytic *L. innocua* improved growth of *A. proteus* population as well as consumption of intact ciliates. Percentage change per hour was − 0.418 ± 0.19%, 0.62 ± 0.26% and 0.78 ± 0.18% in the presence of *L. innocua*-fed, *L. monocytogenes*-fed and control intact *T. pyriformis*, respectively (Fig. 3a, p < 0.005).

Enumeration of intracellular bacteria passed to *A. proteus* by *T. pyriformis* revealed that both pathogenic and saprophytic bacteria might be delivered alive to amoebas (zero time point in Fig. 3b). Meanwhile, other behaviours of pathogenic and saprophytic bacteria were different. A noticeable drop in the number of *L. innocua* was observed within first the 2 h, which was followed by a slight decline. In contrast, the number of intracellular *L. monocytogenes* remained stable over the first 2 h. The decrease in the number of *L. monocytogenes* observed between the 2nd and 6th h changed to resurgence by 24 h (Fig. 3b, Additional file 1: Table S1).

Statistically significant prevalence of amoebas with spherical morphology over pseudopodia-possessing hungry amoebas was observed when pathogenic *L. monocytogenes* were included in the food chain (Fig. 3e, 50th percentile 4.5, 5.5, 4.5 vs 1.25, 0.7, 0.6 for spherical and hungry amoebas at 1, 3, and 24 h, respectively; p < 0.05). Feeding of *A. proteus* with *T. pyriformis* that grazed on saprophytic bacteria caused a slow growth of the amoebal population and prevalence of hungry amoebas with unchanged morphology (Fig. 3f, 1.0, 1.0, 0.45 vs 3.55, 3.6, 3.2, for spherical and hungry amoebas at 1, 3, and 24 h, respectively; p < 0.05).

### LLO is involved in interactions of *L. monocytogenes* with *A. proteus*

To address the role of LLO in interactions of *L. monocytogenes* and *A. proteus* and to evaluate its role in the passage of *L. monocytogenes* along the food chain, we compared two isogenic pairs, each including LLO expressing and non-expressing strains. The first pair was the wild-type *L. monocytogenes* strain EGDe and its deviation with the deletion of the LLO-encoding gene *hly* (EGDeΔhly). The second pair included the *L. innocua* NCTC11288 and its derivative that carried a plasmid pHly/PrfA*. The plasmid pHly/PrfA* carried the LLO-encoding *hly* gene and the prfA* gene, which encodes a constantly active transcriptional regulator PrfA* to provide a constant high-level expression of LLO.

**Fig. 4:**
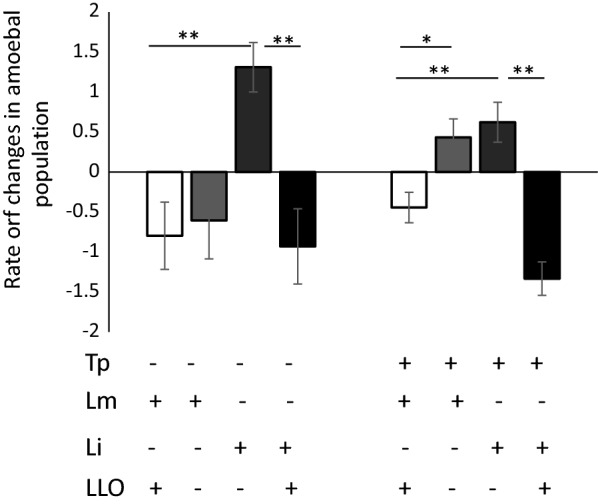
Dependence of dynamics of *A. proteus* population on LLO-production by bacteria. Two isogenic pairs*, L. monocytogenes* strains EGDe (LLO-producing, white) and EGDeΔhly (LLO-lacking, light grey), and *L. innocua* strains NCTC10288 (LLO-lacking, dark grey) and NCTC10288::pHly/PrfA* (LLO-producing, black t) were used to feed *A. proteus* directly or via an intermediate consumer *T. pyriformis*. Cultures were maintained for 24 h. Rates of changes in the number of *A. proteus* are shown. Positive rates mean an increase in the amoebal population while negative rates mean a decrease. Lm: *L. monocytogenes*; Li: *L. innocua*; Tp: *T. pyriformis*; LLO: listeriolysin O; CFU: colony forming units. The data represent a mean ± SD from three independent experiments with the initial *A. proteus* population taken as 100%. *p < 0.05, **p < 0.005

Listeriolysin O expression by *Listerias* caused a decrease in the *A. proteus* population with negative percentage rates independently of whether bacteria were engulfed by amoebas themselves or delivered with *T. pyriformis* (Fig. 4). However, when LLO non-expressing bacteria were compared, a difference between *L. monocytogenes* EGDeΔhly and *L. innocua* was observed that was dependent on whether bacteria were delivered directly or via *T. pyriformis*. Even lacking LLO, *L. monocytogenes* EGDeΔhly bacteria stimulated some decrease in *A. proteus* population. However, no toxic effects were observed when *T. pyriformis* was used as an intermediate link between *L. monocytogenes* EGDeΔhly and *A. proteus*.

### Electron microscopic studies of interactions in the *Listeria*–*Tetrahymena*–*Amoeba* chain

**Fig. 5:**
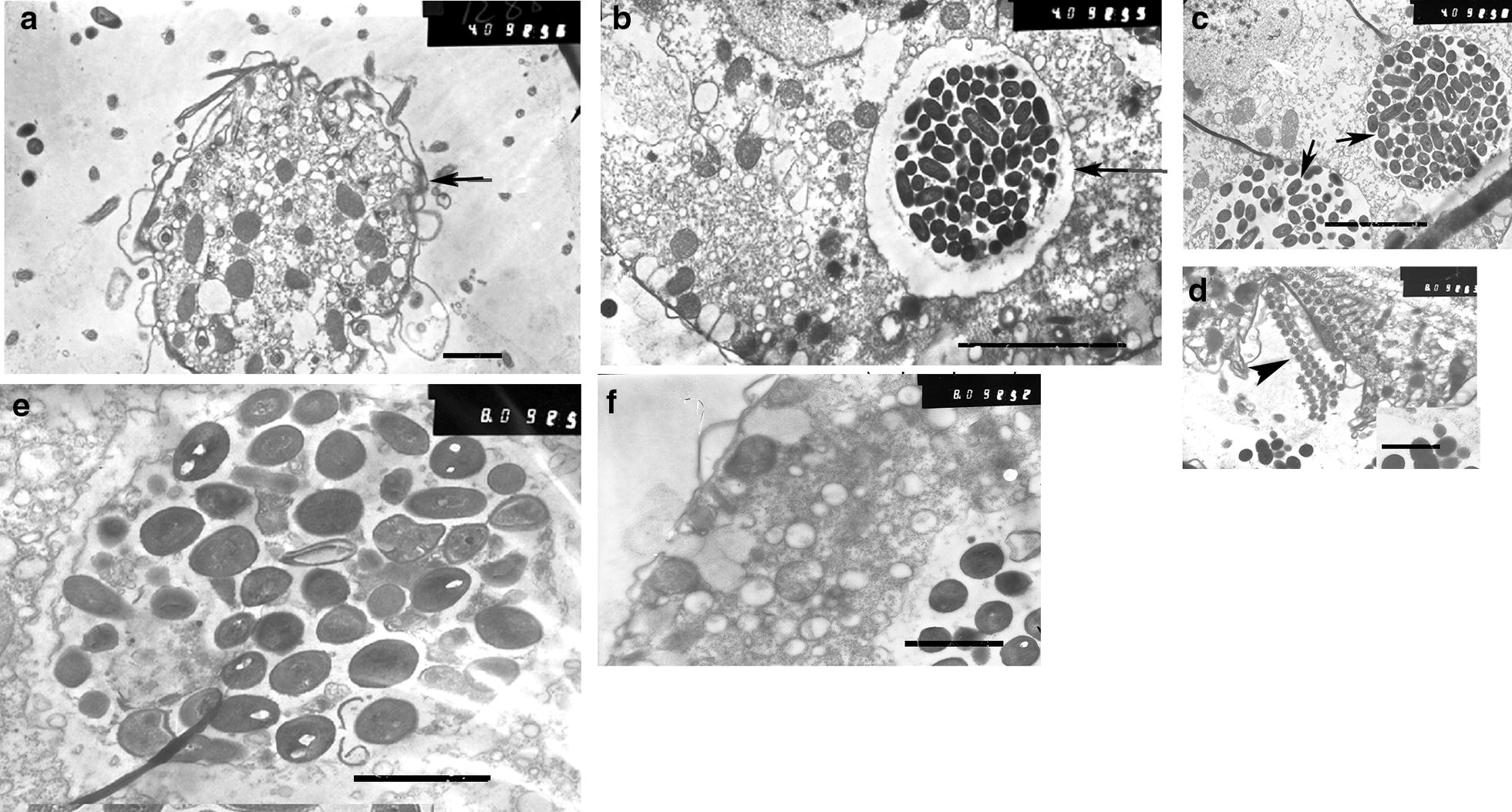
Transmission electron micrographs of *A. proteus* digestive vacuoles. *A. proteus* grazed on intact *T. pyriformis* (**a**), or on *T. pyriformis* fed with *L. monocytogenes* (**b**–**d**) or *L. innocua* (**e**, **f**). Arrows indicate bacteria, arrowhead indicate cilia which are parts of *T. pyriformis*. Bars represent 10 µm (**a**); 5 µm (**b**, **c**), 1 µm (**d**–**f**)

To further address interactions between microorganisms along the food chain, transmission electron microscopy (TEM) was used. Vacuoles that contained the ciliate were observed within *A. proteus* at 3 h post addition of native *T. pyriformis* (Fig. 5a). At the same time point, in accordance with the data of bacterial plating (see Fig. 3a), live bacteria were observed within *T. pyriformis* vacuoles in the *L. monocytogenes*–*T. pyriformis*–*A. proteus* food chain, i.e. when *A. proteus* grazed on *T. pyriformis* which in turn had been fed with *L. monocytogenes* (Fig. 5b, c). Disruption of *T. pyriformis* internal structures might allow for bacteria to occupy the total volume of the phagosome. Further samples revealed vacuoles filled with bacteria and remnants of *T. pyriformis* that supported this idea (Fig. 5d). Bacteria seemed to be still viable and some of them appeared to be dividing. Investigation of *A. proteus* in the *L. innocua*–*T. pyriformis*–*A. proteus* food chain was performed at the same 3 h time interval post addition of *T. pyriformis* to *A. proteus*. It demonstrated phagosomes that contained *T. pyriformis*. Vacuoles filled with live or partly digested bacteria were observed within ciliates (Fig. 5e).

**Fig. 6:**
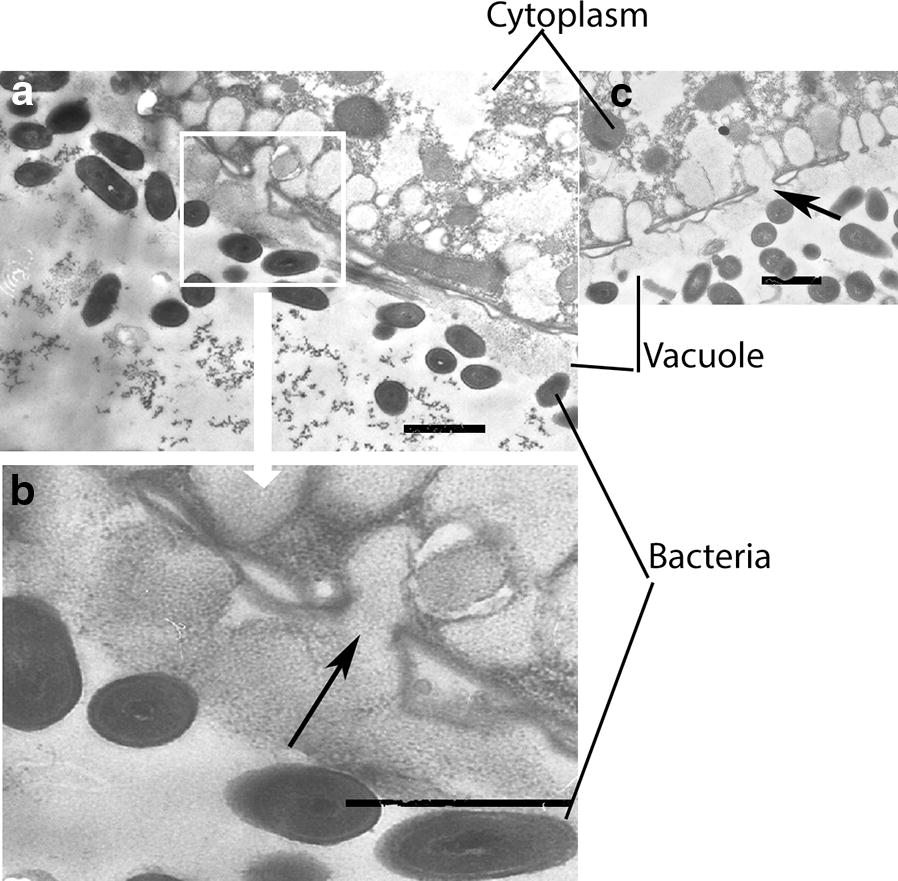
Transmission electron micrographs of *A. proteus* digestive vacuoles with *L. monocytogenes* EGDe (**a**, and its enlarged fragment on **b**) and *L. innocua* pHly/PrfA* (**c**). Membrane perforations are shown with arrows. Bars represent 1 µm

Listeriolysin O is known to be a key factor that contributes to phagosomal disruption in mammalian cells [32]. The multiple bacteria concentrated in phagosomes of *A. proteus* seemed to be associated with perforation of the phagosomal membrane of the protozoa. Bacteria concentrated near the membrane, which lost its integrity (Fig. 6). Perforations were observed when phagosomes contained wild type *L. monocytogenes* (Fig. 6a) and LLO-producing *L. innocua*/pHly/PrfA* (Fig. 6b).

## Discussion

Here, we report the development of an experimental model of the microbial food chain that included virulent or saprophytic *Listeria* spp, the ciliate *T. pyriformis*, and the giant amoeba *A. proteus.* We demonstrated that: (i) the dynamics and physiology of the *A. proteus* population was dependent on whether virulent or saprophytic bacteria were used to feed *T. pyriformis*; (ii) live virulent *L. monocytogenes* might be delivered to *A. proteus* by *T. pyriformis*; and (iii) that passage along the food chain was dependent on *L. monocytogenes* virulence factor LLO.

Since the pioneering work of Ly and Mueller [41], *Tetrahymena* spp. and *Acanthamoeba* spp. have been used as models for the study of *L. monocytogenes* interactions with bacteriovorous protozoa [13, 16, 41–46].

In the present work, in contrast to the small obligatory bacterivorous protists, we used a giant amoeba *A. proteus*. *A. proteus* is a carnivorous protozoan organism though it is capable of digesting bacteria as well [47, 48]. *A. proteus* grazing on both *L. monocytogenes* and its saprophytic counterpart *L. innocua* changed its morphology to a spherical one that is characteristic of prey digesting amoebas. A statistically significant difference was observed in the relative number of amoebas with spherical and pseudopodia-possessing hungry morphology between amoebas that grazed on virulent or saprophytic bacteria. Spherical cells prevailed when amoebas were fed virulent bacteria. Cells with morphology characteristic of hungry amoebas were more numerous when amoebas hunted on saprophytic bacteria. This difference suggested that virulent *L. monocytogenes* slowed down restoration of *A. proteus* morphology from spherical to that possessing pseudopodia with the ability to hunt again. The inability to hunt could be a reason to restrict the size of *A. proteus* population. This suggestion might explain the previously described discrepancy between absence of a short-term cytotoxicity and a global negative effect of *L. monocytogenes* on the protozoan population in conditions of prolonged co-incubation of bacteria and protozoa [13, 16, 41–44, 46, 49].

The food web in terrestrial and aquatic ecosystems includes multiple levels of unicellular consumers. To understand circulation of human pathogens in natural habitats, it is important to study not only mechanisms of bacterial survival within protozoa but also the manner by which pathogenic bacteria are inserted into the microbial food web. From the point of view of matter and energy flow, *T. pyriformis* filled with virulent or saprophytic bacteria represented an equal portion of a meal for *A. proteus*. However, the changes in the *A. proteus* population were dependent on which bacteria was used to feed *T. pyriformis*. Our data are in line with general considerations demonstrating that introduction of parasites noticeably disturbs major parameters of food web functioning [50].

Protozoa are a natural reservoir of pathogenic facultative intracellular bacteria and protect these bacteria from unfavourable environmental factors. They are also an instrument for the selection of virulence traits and the Trojan horses that deliver pathogens to the human body [15]. Our results support the idea of protozoa as a means of delivery of bacteria resistant to digestion to consumers of a higher order and demonstrated a dual role for protozoa as both a “Trojan horse” and “Troy.” The last observations supposed the role of protozoa in the development of traits required for bacterial passage along the food chain.

The developed model of the microbial food chain was used to study a role of the thiol-dependent haemolysin LLO, which is a *L. monocytogenes* key virulence factor. The LLO was chosen because it was shown to contribute to bacterial survival in the presence of *T. pyriformis* and *Acanthamoeba castellanii* [13, 43]. Obtained results suggested that LLO was important for the passage of *L. monocytogenes* along the microbial food chain to higher order consumers. This suggestion was supported by changes in the *A. proteus* population in dependence of whether LLO-producing or non-producing bacteria were used to feed *T. pyriformis*.

## Conclusions

Taken together, our results suggested that pathogenic bacteria, when introduced into the food chain, behave differently from saprophytic bacteria and may possibly disturb the flow of nutrients and energy to consumers at higher orders. The demonstrated dual role of protozoa as “Trojan horse” as well as “Troy” underscores their importance in the evolution of bacterial pathogenic traits in microbial ecosystems.

## Methods

### Microbial strains and growth conditions

Bacterial strains included the wild type *L. monocytogenes* strain EGDe (serovar 1/2a, [35], its derivative EGDeΔhly lacking the LLO-encoding gene *hly* (the strain was kindly provided by Prof. Vazquez-Boland, Univ. Edinburgh), the wild type *L. innocua* strain NCTC 11288 (serovar 6a), and the derivative of the last strain *L. innocua* NCTC 11288::pHly/PrfA [13]. The strain *L. innocua* NCTC 11288::pHly/PrfA* carried a high copy number plasmid pHly/PrfA* which expressed LLO under the positive control of the constitutively active transcriptional regulator PrfA* [13]. Bacteria were kept frozen at − 80 °C. About a week before the experiment started, bacteria were plated onto Brain Heart Infusion (BHI, BD, Sparks, MD, USA) agar and grown at 28 °C. For plasmid-carrying strains, the medium was supplemented with erythromycin (10 μg ml^−1^). An isolated colony was used to inoculate BHI broth. The culture was grown at 28 °C with shaking for 18–20 h. The overnight culture was diluted (1:100) in fresh BHI broth and grown to an OD600 of 1.0. Then, the bacteria were harvested, washed with phosphate-buffered saline (PBS_, resuspended in PBS supplemented with 10% glycerol (1:100 of the initial culture volume), aliquoted, and frozen at − 80 °C. The bacterial concentration was determined by thawing an individual vial and plating serial dilutions on BHI agar. Immediately before the experiment, bacteria were thawed and serial dilutions prepared in PBS were added to the protist culture in the pointed concentration.

Axenic *T. pyriformis* from the Collection of the Gamaleya Institute was maintained on diluted BHI broth (dBHI, BD, 3 g of powder per 1 l of distilled water) supplemented with gentamycin 100 μg ml^−1^, Diflucan 100 μg ml^−1^, and Cyfran 100 μg ml^−1^ at 28 °C. The culture was propagated by a dilution of 1:10 into fresh medium weekly. Ten days before the experiment started, antibiotics were removed from the dBHI medium and the culture of *T. pyriformis* was amplified by a dilution 1:5 every 4 days. The culture was used in the experiment 3 days after the last seeding.

Strains “B” and “CCAP LB1503/4” of *A. proteus*, from the Collection of the Institute of Cytology RAS [51], were used in experiments. Amoebae were cultured on Prescott mineral medium [52] at room temperature according to a standard procedure, and fed with *T. pyriformis* GL ciliates every 48 h [37].

### Amoeba:bacteria co-culture

To evaluate an effect of pathogenic and saprophytic bacteria on *A. proteus*, amoebas were starved for 3 days, then the bacterial culture, prepared as described above, was added to *A. proteus* at a multiplicity of 1000:1 (bacteria:amoeba). The co-culture was maintained in the wet chamber at room temperature for 24 h.

### Microbial food chain modelling

Three-day old *T. pyriformis* culture was diluted up to 10^5^ cells ml^−1^ with fresh dBHI medium. Bacterial culture prepared as described above was introduced into protozoan culture at a multiplicity of 1000:1 (bacteria:protozoa). The co-culture was maintained at 28 °C without agitation for 1 h. Then, protozoa were pelleted by centrifuging at 700 rpm for 10 min, washed with sterile PBS, and resuspended in fresh dBHI medium containing 100 μg ml^−1^ gentamycin to remove extracellular bacteria. One hour later, tetrahymenas were washed with PBS, resuspended in Prescott mineral medium, and added to *A. proteus* at a concentration of 100:1 (tetrahymena:amoeba). The co-culture of *Tetrahymena* and *Amoeba* was maintained in a wet chamber at room temperature. Non-swallowed tetrahymenas were removed at 1 h.

### Protozoan quantification

To count *T. pyriformis*, ciliates were fixed with L buffer (30% acetic acid—70% ethanol) added at a ratio of 1:1. Then, ciliates were counted by microscopy. Amoebas were quantified without fixing using the AxioCam ERc5s (Carl Zeiss) microscope at 100× magnification. Twenty fields of vision were included in analysis from one experiment. Typical fields of vision for different experiments are shown at Figures. The word “hungry” designates pseudopodia-possessing cells with morphology typical for starved amoebas. The word “spherical” designates round-shaped cells that is characteristic of prey digesting amoebas. Rates of changes in amoebal population were calculated as a regression coefficient in the linear regression equation describing changes in amoebal population in each experiment. Then an arithmetic mean and standard error from all experiments were calculated.

### Bacterial quantification

Washed protozoa were lysed with 1% Triton X-100. Decimal serial dilutions were plated onto BHI agar in duplicates and incubated at 37 °C. Bacterial colonies were counted at 24 h.

### Transmission electron microscopy

Transmission electron microscopic (TEM) investigations were performed in general as described in [13]. In short, microorganisms were concentrated, fixed with phosphate-buffered osmium tetroxide, dehydrated in alcohols of increasing concentrations, and embedded in araldite M. Ultrathin sections were produced on an LKB-3 ultratome, and studied in a GEM 100B electron microscope. Up to six sections for one sample were studied.

### Statistics

All experiments were performed using duplicate samples and repeated 3 to 6 times. The data are presented as the mean ± standard deviation (SD) from independent experiments. The data on morphological changes are presented as boxplots from three independent experiments with 20 fields of view each. A two-tailed t-test was used for assessment of statistical significance.

## Supplementary information

**Additional file 1: Table S1.** Number of intracellular bacteria.

## Data Availability

The datasets used and/or analyzed during the current study are available from the corresponding author on reasonable request.

## Abbreviations

LLO: listeriolysin O
TEM: transmission electron microscopy
BHI: brain hear infusion
PBS: phosphate buffered saline
dBHI: diluted BHI
SD: standard deviation
