# Supplementary material for: Experimental Listeria–Tetrahymena–Amoeba food chain functioning depends on bacterial virulence traits
Source: BMC Ecol. 2019 Nov 22;19:47. doi: 10.1186/s12898-019-0265-5 (PMC6874821; doi:10.1186/s12898-019-0265-5)
Supplement: Supplementary file 1 — Additional file 1: Table S1. Number of intracellular bacteria. [file 12898_2019_265_MOESM1_ESM.docx]

Table S1. Number of intracellular bacteria.

| Average  CFU/ml |  | 0^a^ | 2 | 6 | 24 |
| --- | --- | --- | --- | --- | --- |
|  | EGDe | 9800 | 9550 | 7170 | 9603 |
|  | Linn | 8823,529 | 4462,5 | 3945 | 2890,5 |

|  | SE | 0 | 2 | 6 | 24 |
| --- | --- | --- | --- | --- | --- |
|  | EGDe | 1602,775 | 1828,326 | 1631,475 | 511,8815 |
|  | Linn | 2015,496 | 350,1488 | 623,9792 | 1648,725 |

^a^- number of bacteria within 10^5^ T. pyriformis used to feed A. proteus
